# Pharmacophore-Based Study: An In Silico Perspective for the Identification of Potential New Delhi Metallo-β-lactamase-1 (NDM-1) Inhibitors

**DOI:** 10.3390/ph17091183

**Published:** 2024-09-09

**Authors:** Heba Ahmed Alkhatabi, Hisham N. Alatyb

**Affiliations:** 1 Faculty of Applied Medical Science, King Abdulaziz University, Jeddah 21589, Saudi Arabia; khatabiheba@gmail.com; 2 Hematology Research Unit (HRU), King Fahd Medical Research Center (KFMRC), Jeddah 80200, Saudi Arabia; 3 Department of Biochemistry, Faculty of Science, King Abdulaziz University, Jeddah 21589, Saudi Arabia

**Keywords:** antibiotic resistance, drug design, computational drug discovery, virtual screening, MM/GBSA analysis

## Abstract

In the ongoing battle against antibiotic-resistant bacteria, New Delhi metallo-β-lactamase-1 (NDM-1) has emerged as a significant therapeutic challenge due to its ability to confer resistance to a broad range of β-lactam antibiotics. This study presents a pharmacophore-based virtual screening, docking, and molecular dynamics simulation approach for the identification of potential inhibitors targeting NDM-1, a critical enzyme associated with antibiotic resistance. Through the generation of a pharmacophore model and subsequent virtual screening of compound libraries, candidate molecules (ZINC29142850 (Z1), ZINC78607001 (Z2), and ZINC94303138 (Z3)) were prioritized based on their similarity to known NDM-1 binder (hydrolyzed oxacillin (0WO)). Molecular docking studies further elucidated the binding modes and affinities of the selected compounds towards the active site of NDM-1. These compounds demonstrated superior binding affinities to the enzyme compared to a control compound (−7.30 kcal/mol), with binding scores of −7.13, −7.92, and −8.10 kcal/mol, respectively. Binding interactions within NDM-1’s active site showed significant interactions with critical residues such as His250, Asn220, and Trp93 for these compounds. Subsequent molecular dynamics simulations were conducted to assess the stability of the ligand–enzyme complexes, showing low root mean square deviation (RMSD) values between 0.5 and 0.7 nm for Z1, Z2, which indicate high stability. Z2’s compactness in principal component analysis (PCA) suggests that it can stabilize particular protein conformations more efficiently. Z2 displays a very cohesive landscape with a notable deep basin, suggesting a very persistent conformational state induced by the ligand, indicating robust binding and perhaps efficient inhibition. Z2 demonstrates the highest binding affinity among the examined compounds with a binding free energy of −25.68 kcal/mol, suggesting that it could offer effective inhibition of NDM-1. This study highlights the efficacy of computational tools in identifying novel antimicrobial agents against resistant bacteria, accelerating drug discovery processes.

## 1. Introduction

The New Delhi metallo-β-lactamase-1 (NDM-1) is a transferable antibiotic resistance factor that has been discovered to provide enteric bacteria resistance to almost all β-lactams, including the much-discussed carbapenems. This discovery poses a significant risk to human health [1]. NDM-1 has the ability to hydrolyze almost all β-lactam antibiotics [2,3]. Thiosemicarbazone derivatives 19 bg and 19 bh were potent NDM-1 inhibitors that could restore the susceptibility of the meropenem (MEM) against clinical isolates [3]. Almost all β-lactam antibiotics used in clinical settings can be resistant to NDM-1, it is present in a range of enterobacteria, is carried by an easily transmissible plasmid including many additional antibiotic resistance determinants, and may be found in infections that are acquired in the community as well as those that are nosocomial [4]. Derivatives of thiophene-carboxylic acid have demonstrated synergistic antibacterial action when combined with meropenem, and they may inhibit NDM-1 [5].

Prior research has thoroughly recorded the difficulties and advancements in addressing the dissemination and consequences of NDM-1, an enzyme that grants bacteria resistance to many drugs [6]. Studies on NDM-1 have shown that it can break down a variety of β-lactam antibiotics, making them useless. The enzyme’s presence on plasmids allows for easy transmission across bacteria, leading to rapid spread of resistance across many species and habitats [7]. *Klebsiella pneumoniae* and *Escherichia coli* are frequently found to have resistance genes, which are commonly detected in both hospital-acquired and community-acquired illnesses. Previous studies have been conducted to identify inhibitors targeting New Delhi metallo-β-lactamase-1 (NDM-1) and other metallo-β-lactamases, which are responsible for bacterial multidrug resistance [8]. Researchers conducted multistep virtual screening of natural compounds from the NPASS database, using Captopril and NPC120633 as templates. These compounds show potential for inhibiting various metallo-β-lactamase variants and could be valuable in experimental studies to combat antibiotic resistance. Another study conducted where commercially available compounds were screened against clinically relevant β-lactamases (BLs), including CTX-M-15, KPC-2, NDM-1, VIM-2, and P. aeruginosa AmpC [9]. The results highlight preferences of different BLs for specific compound scaffolds, offering insights into potential inhibitor design, with promising compounds identified, including one that enhances imipenem activity against an NDM-1-producing *E. coli* strain [10]. 

The rationale for selecting NDM-1 as the target protein in this investigation is substantiated by its critical function in bacterial resistance to β-lactam antibiotics and its clinical significance [7]. NDM-1 has gained significant recognition for its capacity to hydrolyze an extensive range of β-lactam antibiotics, including carbapenems, which are frequently reserved for the treatment of critical bacterial infections [11]. In this present study, a comprehensive computational approach was employed to identify potential inhibitors of NDM-1ND, a significant enzyme associated with antibiotic resistance. This study utilized a combination of pharmacophore-based virtual screening, molecular docking, and molecular dynamics (MD) simulations to explore the binding interactions between NDM-1 and various compounds. The first step in this study involved the generation of a pharmacophore model based on the structural features essential for binding to NDM-1. This model served as a filter to screen a library of compounds, prioritizing those that exhibited similar pharmacophoric characteristics to known NDM-1 inhibitor. Following virtual screening, the selected compounds underwent molecular docking studies to predict their binding modes and affinities towards the active site of NDM-1. Docking simulations provide insights into the orientation and interactions of the ligands within the binding pocket of the target protein, aiding in the identification of potential inhibitors. To further validate the binding stability and dynamics of the ligand–protein complexes, molecular dynamics simulations were performed. These simulations allowed for the exploration of the conformational changes and fluctuations in the protein–ligand interactions over time, providing valuable information on the stability of the complexes and their suitability as potential inhibitors.

Through this integrative computational approach, this study aimed to identify novel compounds with the potential to inhibit NDM-1 activity, thereby offering new avenues for the development of antimicrobial agents to combat multidrug-resistant bacteria. The findings of this study contribute to the ongoing efforts in drug discovery and the fight against antibiotic resistance, highlighting the significance of computational methods in accelerating the identification of promising drug candidates. 

## 2. Results and Discussion

### 2.1. Protein Structure and Docking

**Figure 1:**
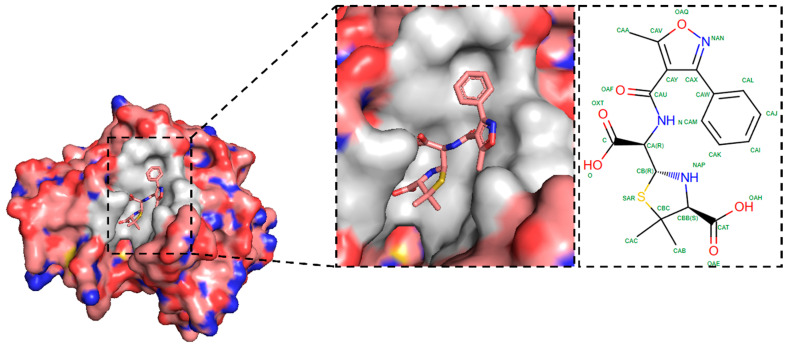
Crystal structure of New Delhi metallo-β-lactamase-1 (NDM-1) bound to hydrolyzed oxacillin (0WO).

In this study, the crystal structure of the protein, the New Delhi metallo-β-lactamase-1 (NDM-1) bound to hydrolyzed oxacillin, was retrieved from the protein data bank (PDB) with the PDB ID: 4EYB. The protein NDM-1 is a highly transferable antibiotic resistance factor that provides enteric bacteria with resistance to almost all β-lactams, including carbapenems, which is a significant risk to human health [1]. β-Lactamase enzymes are categorized into four groups (A–D) according to their DNA sequence similarities [12,13]. Classes A, C, and D enzymes employ an active-site serine as a nucleophile, while class-B enzymes are metallo-β-lactamases (MBLs) that utilize active-site zinc to coordinate a nucleophilic hydroxide for hydrolysis. Figure 1 shows the crystal structure (3D) of NDM-1 bound to hydrolyzed oxacillin (0WO). The electrostatic surface potential of NDM-1 highlights the charged regions around the active site which is visualized using PyMOL (Schrödinger, L., & DeLano, W. (2020). PyMOL. Retrieved from http://www.pymol.org/pymol). A close-up view displays the hydrolyzed oxacillin within the enzyme’s binding pocket, showing its interactions with key residues. The enlarged figure represents a 2D schematic of the hydrolyzed oxacillin, detailing its interactions with the active site, which is identified from RCSB PDB [14].

**Figure 2:**
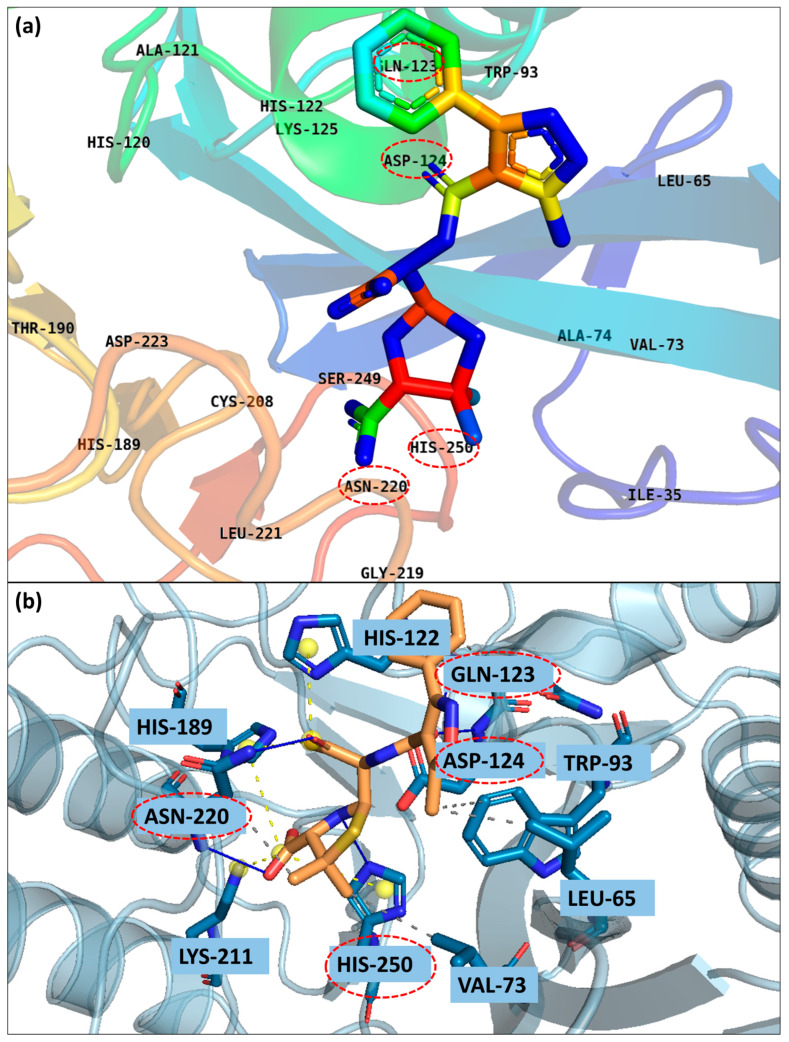
The binding residues have been identified using the PyMOL tool, the binding residues are those located within a 6 Å radius around the known ligand (**a**,**b**) 3D interactions.

The binding site residues around the known bound ligand, hydrolyzed oxacillin (0WO), are shown in Figure 2. The residues responsible for binding were Ile35, Leu65, Met67, Val73, Ala74, Trp93, His120, Ala121, His122, Gln123, Asp124, Lys125, Glu152, Gly153, Met154, His189, Thr190, Cys208, Lys211, Leu218, Gly219, Asn220, Leu221, Asp223, Ser249, and His250. Further, the bonds of the known binder, 0WO, were found through the PLIP (Protein–Ligand Interaction Profiler) server [15]. It was observed that the ligand had hydrogen bonds with the residues Asn220, His250, Gln123 and Asp124. It also showed hydrophobic contacts with Asn220, Val73, Leu65, and Trp93. Further, a salt bridge was observed by the residues His122, His250, Lys211 and His189.

A previous study showed that Zn1 ion showed co-ordination bonds with the residues His120, His122, and His189 and Zn2 ion showed co-ordination bonds with the residues Asp124, Cys208, and His250 [16]. Further, the residues Ile35, Leu65, Met67, Trp93, and Val173 were part of the formation of a large hydrophobic binding surface for hydrophobic interactions with β-lactam R groups [17,18,19]. Asn220 and Lys211 are involved in the substrate recognition and hydrolysis [19]. Gln123 and Asp124 showed hydrogen bonds with oxygen atoms adjacent to hydrophobic β-lactam R groups [17]. Trp93, Gln123, Asp124 and His250 were part of the formation of a hydrophilic hole for substrate binding [20]. These residues were all part of the binding pocket where the known ligand, 0WO. Thus, the grid box was built surrounding this known ligand with the distance of 6 Å from the center.

The use of hydrolyzed oxacillin in this study serves several important purposes despite it not being a direct NDM-1 inhibitor. Firstly, hydrolyzed oxacillin, a product of the beta-lactam antibiotic oxacillin, provides a reference point for understanding the interaction dynamics of NDM-1 with a known beta-lactam structure. This comparison allows us to evaluate whether newly designed inhibitors demonstrate improved binding affinities against a structurally similar but inactive compound. Secondly, the crystal structure of NDM-1 bound to hydrolyzed oxacillin (PDB ID: 4EYB) was utilized as a template for molecular modeling and docking studies, helping to reveal specific interactions within the binding pocket that can guide the design of novel inhibitors. While it is true that hydrolyzed oxacillin may not remain bound in the active site due to its structural changes, its role as a comparative measure is valuable in identifying inhibitors that can effectively compete for binding. This is crucial for developing strategies to use these new inhibitors in combination with beta-lactam antibiotics, ultimately aiming to combat bacterial resistance.

**Figure 3:**
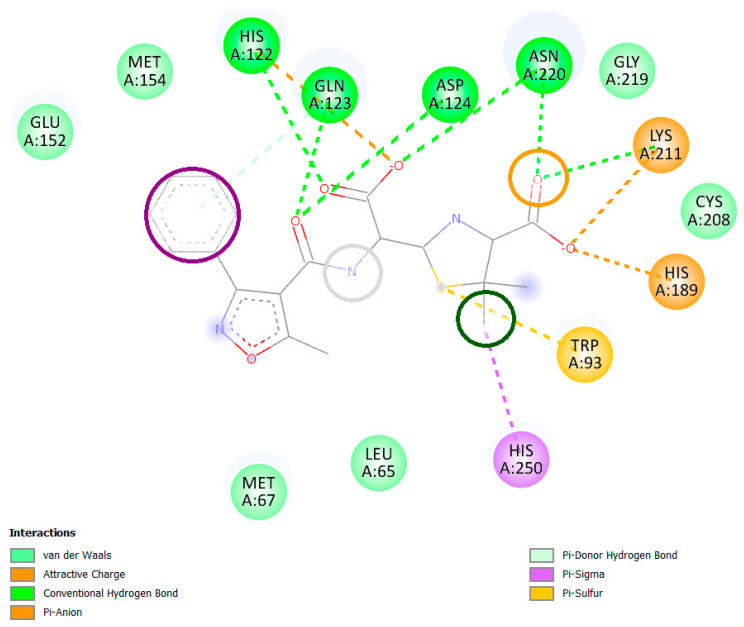
Pharmacophoric features of the ligand–protein (0WO-NDM-1) binding interaction. Aromatic (purple circle), hydrogen acceptor (grey circle), hydrogen donor (yellow circle), and hydrophobic (green circle).

Further, the protein was prepared for molecular docking using Autodock suite and the known ligand, 0WO, was also prepared. Finally, molecular docking was performed using Autodock vina. The best docked pose was used for identifying the significant interaction formed between the known ligand and the NDM-1 protein. Figure 3 shows the interaction plot where the known ligand showed conventional hydrogen bonds with the residues His122, Gln123, Asp124, and Asn220. It also showed Pi-anion with Lys211 and His189, Pi-sulfur with Trp93 and Pi-sigma with His250. These identified key interactions and corresponding pharmacophore features were mapped onto the ligand structure as shown in Figure 3. In Figure 3, the aromatic characteristics (purple circle) were selected to account for any pi-stacking or pi-cation interactions with aromatic residues in the binding pocket. Hydrogen bond acceptors (grey circles) and donors (yellow circles) were integrated to capture key hydrogen bonding capabilities required for molecular recognition. In Figure 3, the nitrogen atom is encircled in grey to denote its potential as a hydrogen bond acceptor in the pharmacophore model. Additionally, hydrophobic features (green circles) were included to represent favorable non-polar interactions between the ligand and hydrophobic regions within the binding site. Although this nitrogen does not directly interact with any amino acid residue in the specific conformation presented, its inclusion in the pharmacophore model is based on its potential to engage in interactions under different binding conformations or dynamic conditions that are not captured in a single static representation. Pharmacophore models are designed to identify potential interaction sites that could contribute to binding affinity and specificity under various physiological conditions or during ligand reorientation. The inclusion of this nitrogen as a hydrogen bond acceptor suggests that it could interact with an amino acid under certain conditions, even if such an interaction is not observed in the specific conformation shown in Figure 3. The primary focus of the pharmacophore model is to capture a broad range of interactions that define ligand efficacy, rather than solely focusing on catalytic residues that are crucial for enzymatic activity.

### 2.2. Pharmacophore

**Figure 4:**
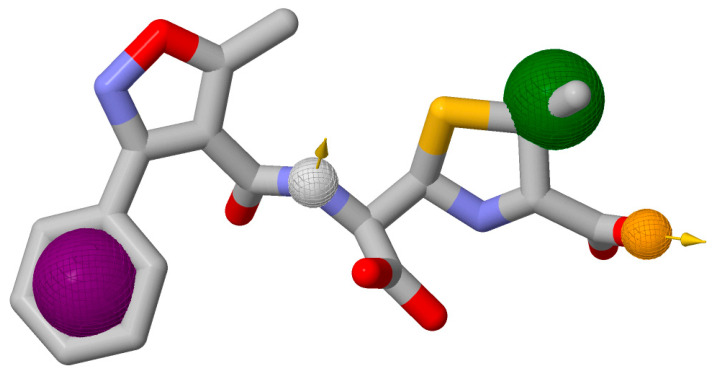
Pharmacophore model used for generating new molecules on ZINCPharmer. It consists of four types of molecular features: aromatic (purple circle), hydrogen acceptor (grey circle), hydrogen donor (yellow circle), and hydrophobic (green circle).

The Mol2 format of native ligand was used in the ZINCPharmer which is accessed on 15 April 2024 and it was loaded as features. Here, four pharmacophores were selected, namely aromatic, hydrogen acceptor, hydrogen donor, and hydrophobic. Here, a maximum RMSD cutoff of 0.3 angstroms was used in the ZINCPharmer and a total of 66 unique hit compounds was generated (Table 1). As shown in Figure 4, the purple sphere indicates an aromatic property of the molecule that can engage in pi-stacking interactions or contribute to electron distribution for target binding. The green sphere shows a hydrophobic component of the molecule that prefers non-polar protein regions. The white sphere with an arrow point to a hydrogen bond acceptor, where a donor could form a hydrogen bond. The yellow sphere with an arrow pointing away is a hydrogen bond donor that can give a hydrogen bond to an acceptor.

In pharmacophore modeling, these features are crucial for identifying and designing compounds with the desired biological activity. All identified features of the molecule are enabled as pharmacophore query features [21]. The image is consistent with the description of the pharmacophore features used in ZINCPharmer search, and the highlighted features will guide the search for new compounds that could potentially inhibit the target protein by mimicking the interactions of the known ligand. The process was validated through the ZINCPharmer server’s built-in protocols, which are designed to ensure the accuracy and reliability of the identified pharmacophore features. The validation process on ZINCPharmer involves cross-referencing the generated pharmacophore features with a large database of compounds to identify those that match the desired features. This process effectively filters out compounds that do not fit the pharmacophore model, thereby validating the model’s ability to identify potential inhibitors. It searches through the database of 18.3 million compounds to find matches to pharmacophore identified [21].

### 2.3. Virtual Screening

The 3D-SDF of a total of 66 compounds screened from pharmacophore were retrieved from ZINCPharmer. The SMILES and 3D structure of the 66 compounds were provided in the Supplementary Sheet S1. Further screening was performed using molecular docking at 100 exhaustiveness and 10 ligand–protein interaction poses predicted for each compound. The binding energy of all 66 ligands were normalized. Normalization of binding scores refers to the adjustment of raw binding scores to a common scale, allowing for more accurate comparison across different compounds by mitigating variability or biases inherent in the original measurements [22]. This process ensures that the binding affinities of various compounds can be directly compared, highlighting those with the most promising interactions. The analysis is then restricted to the top 41 compounds based on these normalized values to focus on those with the strongest binding potential, thereby prioritizing compounds that are most likely to be effective, while also streamlining the research by excluding less relevant data, i.e., according to the normalized binding score, out of 66 compounds, 41 showed better normalized binding score than the control. Table 1 listed the compound ID and their respective normalized scores.

### 2.4. Tanimoto and Clustering

**Figure 5:**
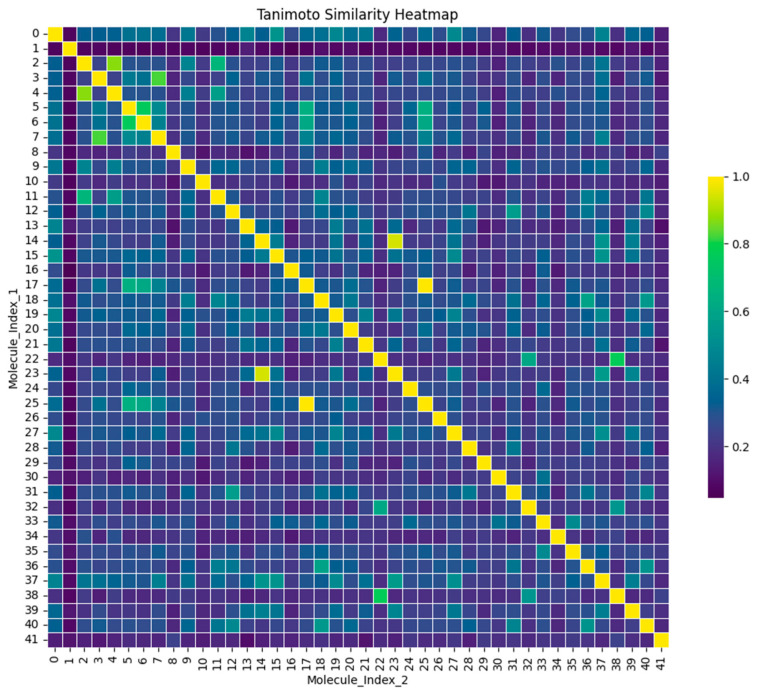
Tanimoto similarity heatmap. The matrix is symmetric, with the diagonal representing the self-similarity of each compound (value of 1.0). The color scale denotes the level of similarity between pairs of compounds, with yellow signifying a high degree of similarity and purple signifying a low degree of similarity.

The Tanimoto similarity was determined for the top 41 compounds and the control. Figure 5 displays a heatmap illustrating the pairwise similarity of 41 chemicals using the Tanimoto similarity index. Cheminformatics uses this index to assess the similarity of structural characteristics of molecules, typically depicted by binary fingerprints where each bit indicates the presence or absence of a specific molecular attribute [23,24]. Both the x-axis and y-axis display the 41 compounds, including the control, indexed from 0 to 41. The value in each cell of the grid indicates the similarity score between the compounds identified by the row and column indices. The similarity score runs from 0 to 1 on the map. A score of 1 (yellow) indicates compounds that are identical or very similar, while a score of 0 indicates compounds that are completely dissimilar. The diagonal from the top-left to the bottom-right represents the comparison of each molecule with itself, resulting in yellow cells indicating a similarity score of 1. Off-diagonal cells display differing levels of similarity across molecules, with colors ranging from green to purple representing decreased similarity.

**Figure 6:**
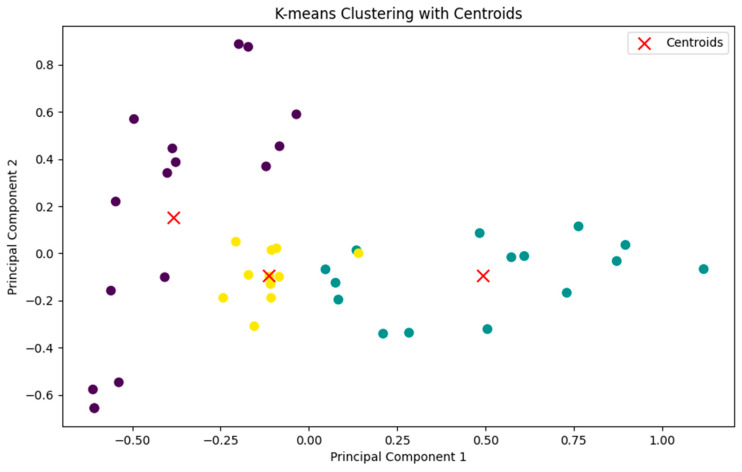
K-means clustering with centroids. Each point represents a compound, and the colors denote the three identified clusters. The red “x” markers indicate the centroids of the respective clusters, which are the mean positions of the data points within each cluster. The centroids serve as representative points for the clusters. The plot shows a clear separation between the three clusters along the principal component axes. Purple represents—Cluster 1, yellow represents—Cluster 2 and green represents—Cluster 3.

Principal component analysis (PCA) was applied to the similarity matrix to reduce the dimensionality of the data to three components. Subsequent K-means clustering on this reduced data formed three distinct clusters, with the intention to group molecules that share similar features. The cluster assignments and centroids are visualized on a scatter plot, where each point represents a molecule, and the centroids are marked with red crosses, which is given in Figure 6. The three compounds nearest to each of the three centroids were found by calculating the Euclidean distance in the reduced dimensionality space to focus on the most representative molecules. The three closest molecules to each centroid as shown in Figure 6 are collected for further analysis. These compounds, ZINC94303138, ZINC78607001, and ZINC29142850, were then used for further detailed analysis, potentially serving as lead candidates for this study.

**Figure 7:**
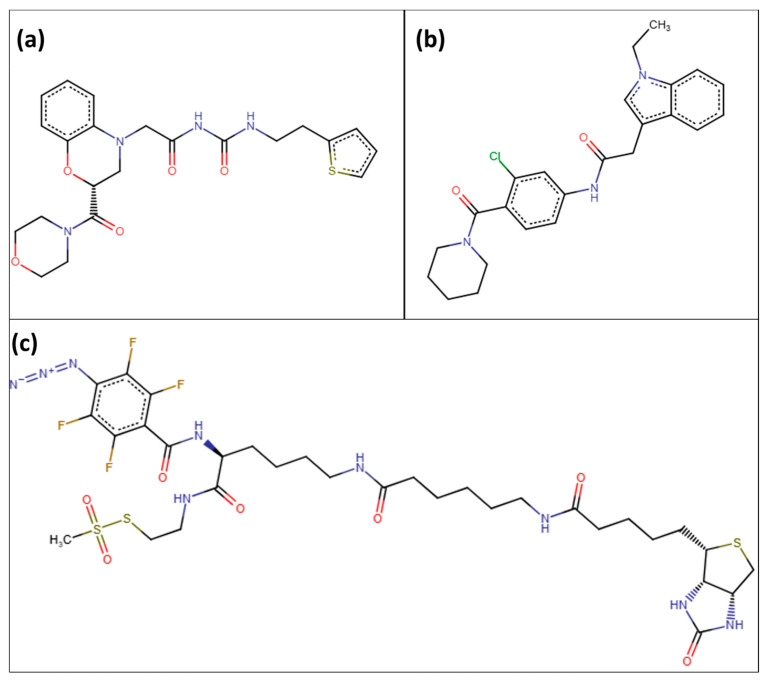
2D structures of the three compounds, (**a**) ZINC29142850 (Z1), (**b**) ZINC78607001 (Z2), and (**c**) ZINC94303138 (Z3).

The control chemical was included in the Tanimoto similarity research and is denoted as index 0 in Figure 5. The degree of similarity between all examined substances and the control was computed and visually shown in the form of a heatmap. Compound 01, despite having no similarity with the other compounds, was deliberately included in this study to enable a comprehensive investigation of chemical diversity. This chemical was retained in the subsequent principal component analysis (PCA) and k-means clustering to assess the behavior of structurally diverse compounds in the dataset. The addition of compound 01 facilitated the identification of several chemical clusters and ensured that the final chosen compounds, which were closest to the cluster centroids, accurately represented the distinct chemical spaces examined in this study. Through this comprehensive method, this study was able to select ZINC29142850 (Z1), ZINC78607001 (Z2), and ZINC94303138 (Z3) as the most representative lead candidates for future investigations. Figure 7 showed the 2D structures of these three compounds.

### 2.5. Interaction Analysis

**Figure 8:**
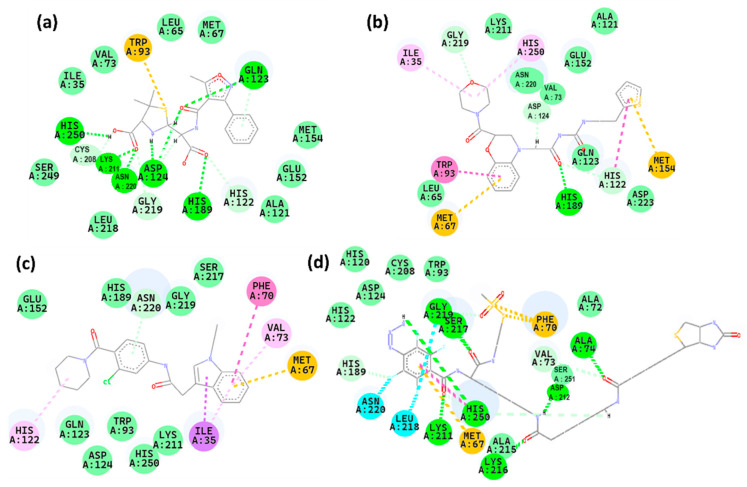
2D representation of the interaction between protein and compounds: (**a**) control, (**b**) Z1, (**c**) Z2 and (**d**) Z3.

Interaction of the protein NDM-1 with the compounds during the docking were analyzed as shown in Figure 8 and listed in Table 2. The control exhibited conventional hydrogen bonds with Gln123, His250, Lys211, Asn220, Asp124, and His189. It also displayed carbon hydrogen bonds with His122 and Gly219, as well as a pi-cation interaction with Trp93. Z1 formed various types of interactions with specific amino acids in the protein: conventional hydrogen bonds with His189; carbon hydrogen bonds with His122, Gly219, Asp124; Pi-alkyl interactions with Ile35, His250; Pi-Pi T-shaped interaction with Trp93; Pi-cation interaction with Met67, Met154. Z2 formed various interactions with different amino acids in the protein: a carbon-hydrogen bond with Asn220, a Pi-Pi T-shaped interaction with Phe70, Pi-alkyl interactions with His122 and Val73, a Pi-cation interaction with Met67, and a Pi-sigma interaction with Ile35. Z3 formed conventional hydrogen bonds with Gly219, Ser217, His250, Lys211, Kys216, Asp212, and Ala74. It also formed carbon hydrogen bonds with His189 and Val73, Pi-cation interactions with Met67 and Phe70, and halogen bonds with Asn220 and Leu218. As shown in Table 2, the interaction of the control with His250 and His189 was found common in both Z1 and Z3 while interaction with His122 was found in common in Z2. Thus, all these compounds showed significant interactions with the protein NDM-1. Further, the 3D interactions were shown in the Supplementary Figure S2. It was observed that all the three hit compounds were bound to the binding site similar to the control. The control had a binding score of −7.30 kcal/mol, whereas the compounds Z1, Z2, and Z3 exhibited higher performance with binding scores of −7.13, −7.92, and −8.10 kcal/mol, respectively.

A previous study showed that ZINC05683641 had key interactions with Ile35, Met67, Val73, Trp93, Cys208, Asn220, and His250, predominantly in the catalytic activity region, indicative of competitive inhibition [25]. Another study showed that ZINC84525623 forms stable complexes via electrostatic and hydrophobic interactions, influencing the catalytic efficiency of NDM-1 [26]. Additionally, another study showed that PHT427 interacts through key amino acid residues such as Asn220, Asp124, and Gln123 [27].

Interactions with residues like His250, Asn220, and Trp93 are crucial across different compounds for mediating interactions with NDM-1. Similar to the inhibitors discussed in previous studies, the compounds in this study interact at critical catalytic and structural regions of NDM-1, which could suggest potential pathways for competitive or non-competitive inhibition. Further to study the stability and flexibility of the compounds with NDM-1, molecular dynamics simulation was performed for 300 ns.

### 2.6. ADMET Properties

The ADMET analysis of the compounds Z1, Z2, and Z3 provides valuable information on their potential as drug candidates, especially when assessed against Lipinski’s Rule of Five. This rule is often used to forecast the likelihood of pharmaceuticals being absorbed orally. Table 3 listed the ADMET properties of the three selected compounds. Z3 has a markedly greater molecular weight (839.94 Daltons) in comparison to Z1 (458.53 Daltons) and Z2 (423.94 Daltons), surpassing the suggested threshold of 500 Daltons as per Lipinski’s Rule of Five. Z3’s potential bioavailability may be hindered due to its larger molecular weight, which often indicates lower absorption and penetration rates. In addition, Z3 exhibits 6 hydrogen bond donors and 14 hydrogen bond acceptors. These characteristics can improve the ability to bind to biological targets. Z3 also possesses an iLOGP value of 0, which signifies that it lacks hydrophobic properties. This may decrease the probability of unintended effects. In addition, Z3 has a PAINS alert, which indicates a potential for generating false positive results in screening assays. This might potentially complicate the process of developing the medicine. On the other hand, Z1 and Z2 exhibit more advantageous characteristics. Both compounds possess molecular weights that fall inside the permissible range—458.53 Daltons for Z1 and 423.94 Daltons for Z2—suggesting a higher probability of being absorbed by the body. In addition, they closely follow Lipinski’s guidelines for hydrogen bond donors and acceptors. Z1 has 2 donors and 5 acceptors, whereas Z2 has 1 donor and 2 acceptors. This indicates that they likely have favorable membrane permeability and bioavailability. The iLOGP values of 1.49 for Z1 and 3.69 for Z2 provide more evidence of their potential as medications that can be taken orally. Z2 is somewhat more hydrophobic, which is normally advantageous for the passive movement across cell membranes. Crucially, both Z1 and Z2 do not exhibit any Lipinski violations or PAINS alarms, which establishes them as robust contenders for further advancement.

All of the compounds demonstrated acceptable solubility. Furthermore, in accordance with the Globally Harmonized System of Classification and Labelling of Chemicals (GHS), these chemicals have been categorized as toxicity class 4, as authorized by the Hazard Communication Standard (HCS). The classification, which suggests that the substances does not pose a risk if ingested and are within permissible thresholds for further advancement.

### 2.7. Molecular Dynamics Simulation (300 ns)

**Figure 9:**
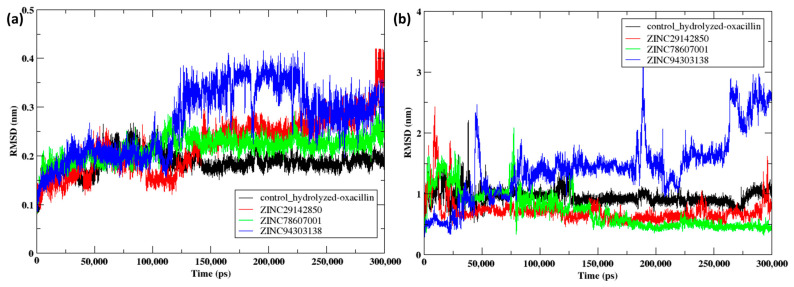
Post MD simulation analysis NDM-1 when bound to the compounds Z1, Z2 and Z3 along with the control. (**a**) RMSD of the protein Cα atoms and (**b**) RMSD of the ligands during the 300 ns MD simulation.

The root mean square deviation (RMSD) of the NDM-1 protein was calculated over a 300 ns trajectory when interacting with these substances and a control. The protein exhibited a steady conformation when bound to the control, as depicted in Figure 9a, with a constant RMSD of 0.2 nm during the entire 300 ns. The data exhibited variations during the initial 100 ns but then remained constant until 300 ns, suggesting that the ligand might have stabilized the protein. Protein-bound Z2 exhibited a consistent RMSD trend over 100 ns, with fluctuations that peaked at 0.25 nm and then stabilized during the last 200 ns. The protein attached to Z1 exhibited fluctuations over the first 150 ns, which then stabilized around 0.25 nm until 270 ns. Further the RMSD of the protein has risen to 0.4 nm over the past 30 ns.

The ligand RMSD values shown in Figure 9b from the 300 ns molecular dynamics simulations reveal considerable differences in the stability and binding kinetics of the ligands with NDM-1. The control compound first showed variations but eventually stabilized with a RMSD of 1 nm for most of the simulation, indicating a consistent interaction after the initial adjustment. Z1 first showed variations but eventually attained improved stability with a decreased RMSD of 0.7 nm, suggesting a more precise and consistent binding. The RMSD analysis of Z3 during the 300 ns molecular dynamics simulation suggests that the ligand maintained a strong and stable interaction with the protein. Initially, the RMSD rose slightly to 2.5 nm, indicating conformational adjustments, but it stabilized at 1.5 nm from 75 ns to 250 ns, reflecting consistent binding. Post 250 ns, the RMSD fluctuated to 2.5 nm, potentially indicating a shift in binding site or conformation. However, the ligand remained bound, highlighting its ability to adapt and maintain strong interactions, which is crucial for effective binding in varying protein conformations. Z2exhibited similar stability to Z1, with a consistently low RMSD, suggesting a steady interaction during the simulation. Comprehending the RMSD patterns is crucial for understanding the dynamic behavior of these compounds within the protein environment and their impact on their potential as NDM-1 inhibitors.

Z1 and Z2 shows as low RMSD values suggest consistent interactions throughout the simulation. A similar study identified ZINC84525623 forming a stable complex with NDM-1 showing lower RMSD values, indicating a robust and persistent binding during molecular dynamics simulation [27]. The study examined phytochemicals such as monodemethylcurcumin, butein, and rosmarinic acid as possible NDM-1 inhibitors [28]. These compounds showed stable interactions with low RMSD values during simulations, similar to Z1 and Z2 as noted in the analysis. The in-depth examination of T008 and T016 compounds shows their robust and enduring binding, with little RMSD values noted across simulations, aligning with the characteristics of your more stable molecules [29]. These studies highlight the importance of keeping low RMSD values when forecasting the stability and effectiveness of putative inhibitors in biological environments.

Consistent and low RMSD values, such as those observed with Z1 and Z2 in this study, suggest that they are strong and possibly effective inhibitors of NDM-1. Higher fluctuations shown with Z3 may indicate less dependable interactions, potentially affecting its efficacy as an inhibitor. This aligns with findings from earlier research, where increased RMSD values often indicate less stability and efficacy in vivo.

**Figure 10:**
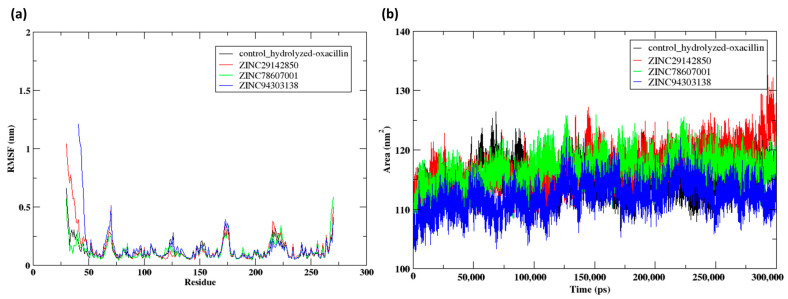
Post MD simulation analysis NDM-1 when bound to the compounds Z1, Z2 and Z3 along with the control, (**a**) RMSF of the protein Cα atoms and (**b**) SASA of the protein during the 300 ns MD simulation.

Root mean square fluctuation (RMSF) data from Figure 10a illustrate the dynamic behavior of NDM-1 when interacting with different ligands, providing valuable insights on the flexibility of individual residues in the protein. The terminal residues Glu30-Arg32, including the control, have higher RMSF values ranging from 0.3 to 1.3 nm, suggesting substantial flexibility in these residues. These protein areas appear to have less restrictions and are likely to undergo more structural changes when a ligand binds. High RMSF values at residue Pro175 were observed for both the control and Z2, particularly at 0.3 nm, indicating that this residue may be a hotspot for dynamic behavior when interacting with both ligands. Changes in key residues may impact the stability of interactions between the ligand and protein, thereby altering the effectiveness of inhibitors.

This detailed RMSF analysis is crucial for understanding which parts of the protein are more flexible or rigid when interacting with inhibitors and can guide the development of more effective drugs by targeting regions that significantly alter the protein dynamics upon binding. Such insights are valuable for refining drug design strategies to enhance binding affinity and specificity by stabilizing key flexible regions of the target protein.

The solvent-accessible surface area (SASA) of NDM-1 shown in Figure 10b offers valuable information regarding the conformational exposure of the protein to the solvent when it is bound to different compounds, including the control. The SASA trends that you delineated exhibit a consistent pattern under the majority of circumstances, commencing at approximately 110 nm^2^ and rising marginally to 120 nm^2^ throughout the simulation, with negligible fluctuations. This observation implies that the protein maintains a relatively stable conformational state in general, as evidenced by the negligible surface area expansions that occur upon ligand binding. The increase in SASA to 130 nm^2^, which is unique to Z1, during the final 5 ns of the simulation, is intriguing. This observation may suggest a unique conformational alteration or an alternative binding mechanism in comparison to the other compounds, potentially exerting a more pronounced impact on the protein’s dynamics and interaction with its surroundings. This significant rise in SASA could indicate that Z1 induces a unique structural change, potentially leading to an alternative binding mechanism that affects the protein’s dynamics and interaction with its environment. Such a conformational shift might imply a more pronounced impact on the protein’s function, making this compound particularly interesting for further study in the context of therapeutic inhibition. This consistent pattern in SASA for the majority of compounds, excluding Z1, indicates that although the binding of these compounds does not substantially alter the protein’s exposure to the solvent, particular interactions may still induce substantial conformational changes.

**Figure 11:**
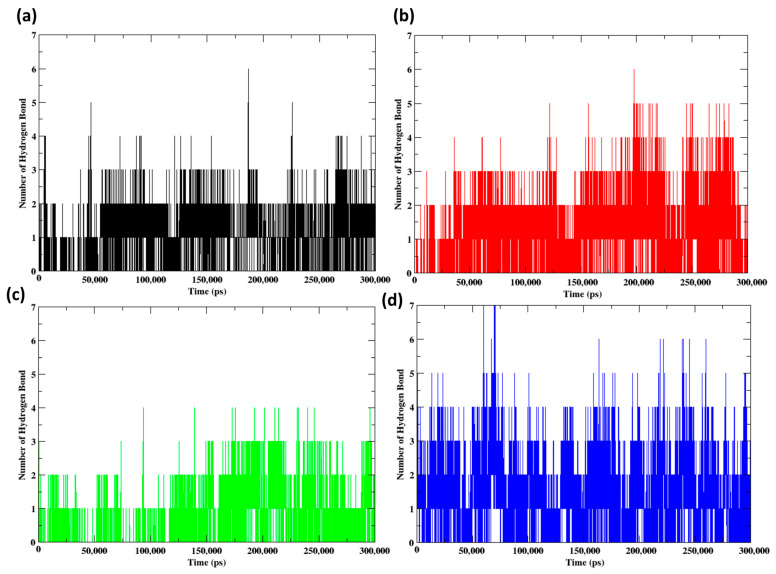
Hydrogen bonds formed between protein and the ligands for (**a**) control, (**b**) Z1, (**c**) Z2 and (**d**) Z3 during the 300 ns MD simulation.

The hydrogen bond analysis (Figure 11) conducted during a molecular dynamics simulation lasting 300 ns offers significant insights into the intermolecular motion of NDM-1 with a control compound and the ligands Z1, Z2, and Z3. A stable pattern of predominantly two hydrogen bonds, with occasional fluctuations to three, was observed in the control throughout the simulation, indicating a consistent interaction with the enzyme. Z1 exhibited comparable stability in hydrogen bonding, sustaining two to three bonds between 200 and 280 ns while experiencing transient periods of increased bonding (up to 4). This may be the result of momentary conformational alterations that improve its affinity for the enzyme. On the contrary, Z2 demonstrated a generally lower number of hydrogen bonds (1-2), with occasional increases to three. This suggests that its interaction with NDM-1 may be weaker or more variable. In contrast, Z3 exhibited more robust activity throughout the initial 50 ns of the simulation, consistently forming 3–4 hydrogen bonds and culminating at 5–6, indicating a potentially more potent inhibitory interaction. This could be due to the specific structural features of the ligand, such as the presence of multiple hydrogen bond donors or acceptors that are well-aligned with complementary residues in the binding site. Additionally, the ligand’s initial binding conformation may be particularly favorable for hydrogen bonding, placing its functional groups in close proximity to those in the binding site. The aforementioned dynamics underscore the potential of molecular dynamics simulations in evaluating the stability and effectiveness of enzyme inhibitors, which are both critical for their advancement as therapeutic substances.

**Figure 12:**
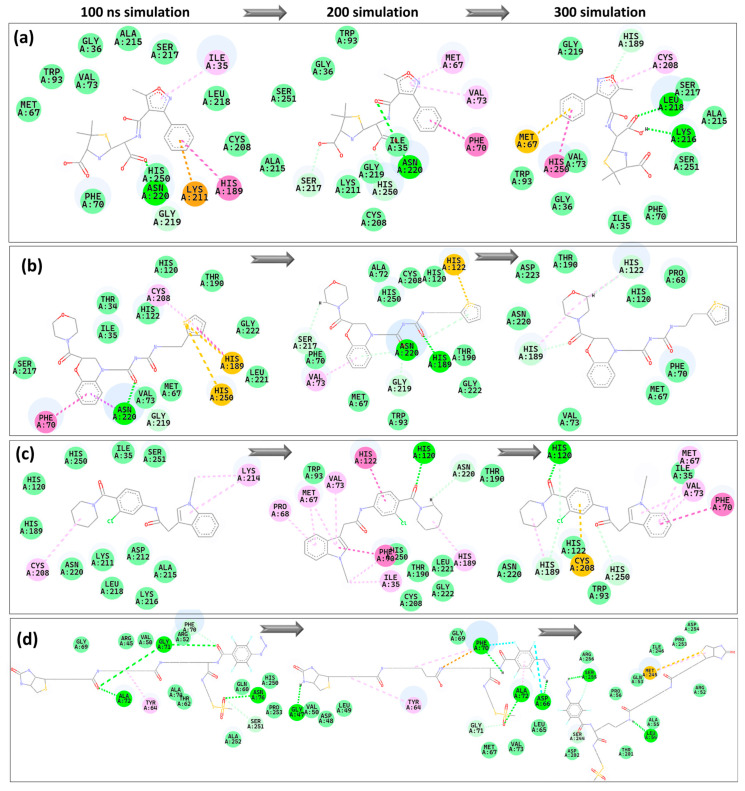
2D representation of the interaction between protein and the ligands: (**a**) control, (**b**) Z1, (**c**) Z2 and (**d**) Z3 during the 100 ns, 200 ns and 300 ns trajectories.

Figure 12 showed the interaction between the protein and ligands during the 300 ns. The control consistently establishes a conventional hydrogen bond with Asn220 at 100 ns and 200 ns. At 300 ns, the contact extends to involve hydrogen bonds with Leu218 and Lys216, indicating a change or widening in the interaction areas. The control shifts from carbon-hydrogen bonds with Ser217 and His250 at 200 ns to hydrophobic interactions with these residues by 300 ns, suggesting a potential alteration in the binding conformation or dynamics. Z1 consistently engages in conventional hydrogen bonding interactions with Asn222 at 100 ns and 200 ns, and also forms a conventional hydrogen bond with His189 at 200 ns. At 300 ns, the bond with His189 transitions to a carbon-hydrogen bond, while a new carbon-hydrogen bond forms with His122, indicating evolving interaction patterns. Z2 does not exhibit hydrogen bonding at 100 ns, but it forms a traditional hydrogen bond with His120 by 200 ns, which persists at 300 ns. By 300 ns, it establishes carbon-hydrogen bonds with His189 and His250, following interaction patterns observed in both the control and Z1, indicating shared binding motifs. Z3 exhibited a dynamic interaction profile, evolving over time with different typical hydrogen bonds forming at various stages of the simulation. Z3 at 100 ns had similar hydrogen bonds including Gly71, Ala72, and Asn76. By 200 ns, the interactions involved Gly40, Phe70, Ala72, and Asp66. At 300 ns, more changes occur involving hydrogen bonds with Leu54 and Ser255, suggesting a very dynamic interaction environment. These observations suggest that Z3 engages in a highly dynamic binding process, which could be advantageous in accommodating protein conformational changes.

Common residues like Asn220, His189, and His250 are crucial in NDM-1 interactions with inhibitors, as shown in multiple investigations. Asn220 frequently participates in important hydrogen bonding, showing its critical location inside the active site of NDM-1. His189 and His250 are commonly focused on due to their catalytic importance, frequently establishing dynamic hydrogen and carbon hydrogen bonds that change across simulations. The interactions highlight the significance of these residues in stabilizing the enzyme-inhibitor complex and improving inhibitor effectiveness, consistent with previous research indicating that such interactions are crucial for successful binding [28,30]. Insights like this are essential for improving the design of NDM-1 inhibitors by concentrating on the frequent and critical interaction sites.

### 2.8. Principal Component Analysis

**Figure 13:**
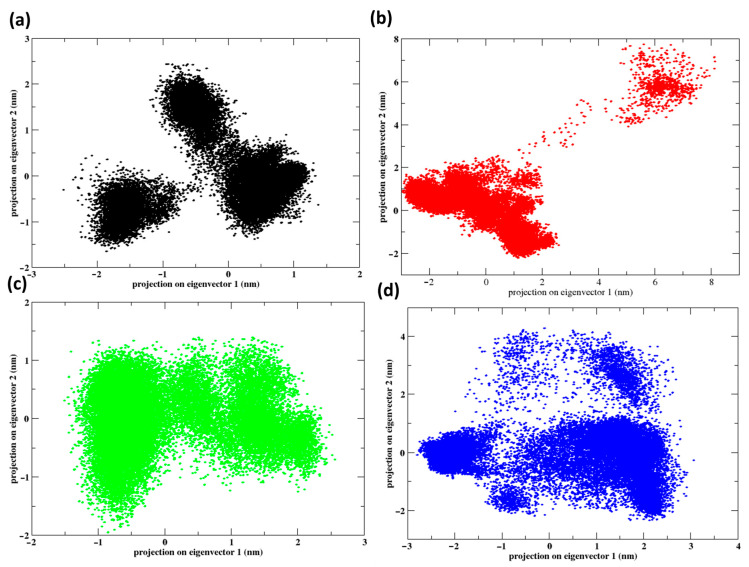
Principal component analysis of the protein bound to the ligands and control during the 300 ns simulation: (**a**) control, (**b**) Z1, (**c**) Z2 and (**d**) Z3.

Figure 13 displays the principal component analysis (PCA) of protein dynamics when interacting with various ligands and a control throughout a 300 ns molecular dynamics simulation. The plots show how the protein’s conformations are spread out over the first two primary eigenvectors, demonstrating the impact of various ligands on the protein’s flexibility and stability. Control indicates the presence of two separate clusters, implying that the protein takes on two primary conformations. The bimodal distribution suggests a notable flexibility in conformation when control in present. Z1 shows a wide, somewhat spread-out cluster along the eigenvector 1 axis, suggesting that this ligand might encourage or support a broader variety of conformations in comparison to the control. The distribution along eigenvector 2 is thinner, indicating that the ligand permits flexibility in one dimension but restricts it in another. Z2 has a denser and more centralized cluster than Z1, indicating stricter control over conformation when this ligand is attached. Z2 ‘s compactness suggests that it can stabilize particular protein conformations more efficiently. Z3 displays a significant dispersion mostly along eigenvector 1, like Z1, but also exhibiting a broader distribution along eigenvector 2. This pattern suggests that Z3 may cause or support a wider range of conformations than those observed with Z1.

### 2.9. Free Energy Landscape

**Figure 14:**
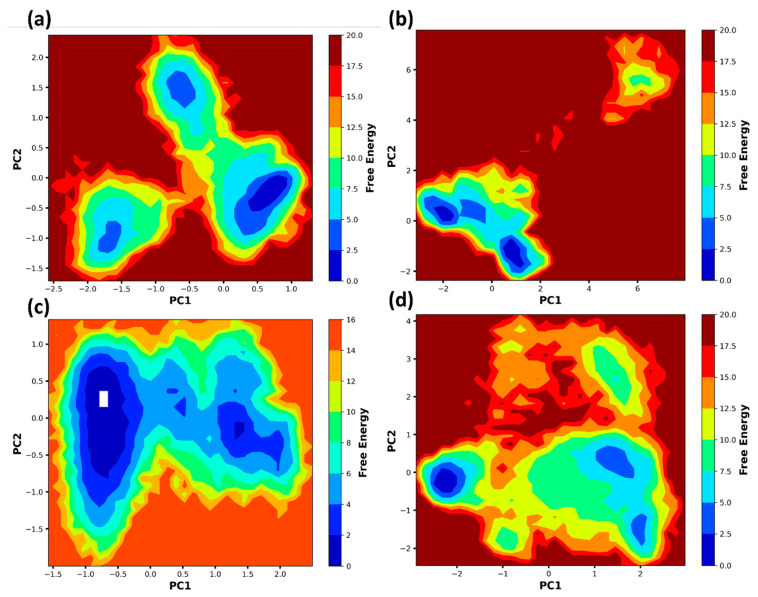
Free energy landscape of the protein bound to the ligands and control during the 300 ns simulation: (**a**) control, (**b**) Z1, (**c**) Z2 and (**d**) Z3.

Figure 14 displays the free energy landscapes of NDM-1 protein interactions with different ligands, analyzed using principal component analysis (PCA). The plots show the changes in free energy along the principal components (PC1 and PC2), which are main axes of motion obtained from the molecular dynamics simulation data.

Control exhibited several distinct and well-defined free energy minima or basins. The separate areas indicate that the protein in its original state can consistently adopt multiple conformations. Z1 displays a fragmented landscape with fewer and less distinct low-energy basins. It indicates that this ligand may cause a less stable conformational state in the protein than the control. Z2 displays a very cohesive landscape with a notable deep basin. This suggests a very persistent conformational state induced by the ligand, indicating robust binding and perhaps efficient inhibition. Z3 displays an intricate terrain with numerous basins of different depths. The ligand causes multiple stable conformations in the protein, possibly indicating flexible binding mechanisms or interactions.

The effect of each ligand on the protein’s structural flexibility and stability can greatly impact its therapeutic efficacy, determining its ability to inhibit or regulate the protein’s function. Z2 demonstrates the most stable conformation. This plot shows a single, deep energy basin, indicating that the protein adopts a highly stable configuration when bound to this ligand.

### 2.10. Binding Free Energies

**Figure 15:**
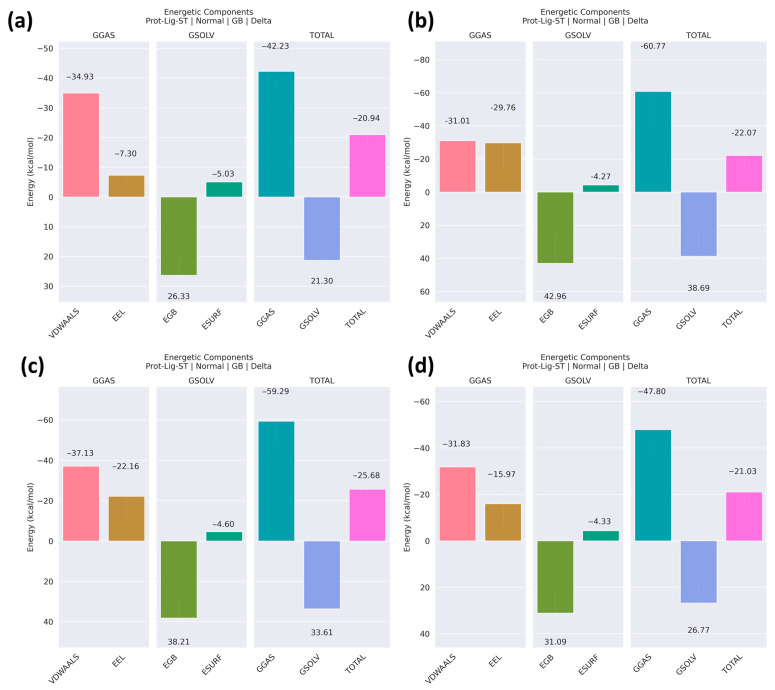
Binding free energy of the complexes of the protein NDM-1 and ligands: (**a**) control, (**b**) Z1, (**c**) Z2 and (**d**) Z3.

The binding free energy values from Figure 15 provide insights into the affinity and stability of the interactions between NDM-1 and various ligands in a simulated environment. The control molecule shows a significant interaction with a binding free energy of −20.94 kcal/mol, establishing a baseline for comparison. Z1 has a slightly higher binding free energy of −22.07 kcal/mol compared to the control, indicating a stronger affinity for the protein. This suggests that Z1 has unique molecular characteristics or engages in more effective interactions with the protein, possibly due to favorable binding pocket complementarity or efficient molecular alignment. Z2 demonstrates the highest binding affinity among the examined compounds with a binding free energy of −25.68 kcal/mol. This significant negative value reflects robust and potentially durable interactions with the protein, highlighting its potential as a strong candidate for further research and drug development. Z3, with a binding free energy of −21.03 kcal/mol, although still more favorable than the control, shows lower affinity compared to Z2. However, the interaction of Z3 with the protein were comparable to the compound Z1. Therefore, despite the observed fluctuations in the RMSD graph, the overall binding stability and dynamic interaction profile justified the inclusion of Z3 in this study.

The Supplementary Table S2 shows the standard deviation (SD) and standard error of the mean (SEM) for various energy components—EEL, EGB, ESURF, GGAS, GSOLV, and TOTAL—calculated using the MMGBSA method for the control, Z2, and Z3 compounds. The table shows the variability in the energy calculations, with notable differences observed across the different ligands. For instance, Z2 exhibits higher variability in the electrostatic energy (EEL) and gas-phase energy (GGAS) components, as reflected by its larger SD and SEM values compared to the control. In contrast, Z3 shows relatively higher variability in its solvent accessible surface area (ESURF) component. The overall binding free energy variability (TOTAL) is lowest for the control and highest for Z3, indicating that the binding stability might differ significantly among these compounds. These error metrics are crucial for assessing the reliability and consistency of the MMGBSA results in the context of molecular binding studies.

## 3. Material and Methods

### 3.1. Protein Structure and Molecular Docking

In this study, the crystal structure of the protein, the New Delhi metallo-β-lactamase-1 (NDM-1) bound to hydrolyzed oxacillin (0WO) was retrieved from the protein data bank (PDB) [14] with the PDB ID: 4EYB [1,31]. This crystal structure of NDM-1 in complex with antibiotics offers insights into its interaction with β-lactam drugs, including carbapenems, and how it confers resistance to these antibiotics [1,32]. OWO is important as it serves as the key substrate that demonstrates the enzyme’s ability to confer resistance to beta-lactam antibiotics, including penicillins like oxacillin [33].

In this study, hydrolyzed oxacillin was employed as a reference compound to understand the interaction dynamics of NDM-1 with a known beta-lactam structure. Although not a direct inhibitor, hydrolyzed oxacillin, derived from the beta-lactam antibiotic oxacillin, provided a valuable template for molecular docking studies. The crystal structure of NDM-1 bound to hydrolyzed oxacillin (PDB ID: 4EYB) was utilized to reveal specific interactions within the NDM-1 binding pocket, guiding the design of novel inhibitors. Docking studies with alternative potential inhibitors, such as L-captopril (PDB ID: 4EXS) and bisthiazolidine, revealed significantly weaker binding energies (−4.8 and −4.5 kcal/mol, respectively) as shown in Supplementary Table S1, making them less suitable for comparison. Therefore, hydrolyzed oxacillin was selected as a reliable reference for evaluating the binding affinities of the newly designed inhibitors in this study. This approach is critical in the ongoing effort to develop effective NDM-1 inhibitors that can be used alongside beta-lactam antibiotics to combat bacterial resistance.

Grid box made around native ligand residues using Autodock (version-1.5.7) [34] and configuration file was prepared. The Autodock tool was used to create a grid box around the native ligand residue, with a distance of 6 Å. The dimensions of the grid at the center were −4.11 Å, 9.34 Å, and 26.26 Å for the x, y, and z axes, respectively. The size of the grid was 18 Å × 16 Å × 16 Å for the x, y, and z axes correspondingly. Furthermore, precise docking parameters were established to maximize the outcomes. Docking performed at 100 exhaustiveness and 10 ligand–protein interaction poses predicted. Protein–ligand interaction analysis using Biovia Discovery Studio. Subsequently, the protein that was identified underwent molecular docking using Autodock Vina [35].

### 3.2. Pharmacophore

The pharmacophores based on ligands are generated utilizing the ZINCpharmer tool (version 1.3) [21]. The ZINCpharmer is an internet-based application that employs the Pharmer pharmacophore algorithm to search the ZINC database for available compounds. The precise spatial arrangement of the fundamental characteristics of biological interaction, such as charged pharmacophores or hydrophobic aromatic features, is regulated by essential functional groups [36]. Here, 3-point pharmacophores were generated using the known binder, hydrolyzed oxacillin (0WO). As features, a Mol2 file containing the native ligand was loaded, and four pharmacophores—aromatic, hydrogen acceptor, hydrogen donor, and hydrophobic—were selected. By employing a maximum RMSD cutoff of 0.3 angstroms, ZINCPharmer successfully produced unique hit compounds.

### 3.3. Virtual Screening

All 3D-SDF structures were downloaded from ZINCPharmer. All structures were minimized using an open-babel tool [37] with MMFF94 forcefield in 2500 steps [38]. Finally, docking performed at 100 exhaustiveness and 10 ligand–protein interaction poses were predicted for each compound. The same parameters and protocol were applied as mentioned in Section 2.1. The binding scores of all ligands have been normalized and ranked according to the normalized scores. The normalized scores were calculated with the equations below:

(1) average=Average of(Binding Energy/Top Binding Energy)

(2) Normalized Score=Top Binding Energy×average

### 3.4. Tanimoto Similarity and Clustering

The compounds that interacted more effectively than the control were selected for additional screening based on the highest binding energy. Using the RDKit program (version 2023.03.3) [39] Tanimoto similarity [40] and additional comparisons were performed on the screened compounds. For clustering within the sklearn package (version - 1.2.2) of Python (version 3.10.8) [41], the K-means function of the cluster module was applied [41]. All of the plots were generated utilizing the matplotlib module in Python [42]. The stepwise screenshot of the steps has been shown in the Supplementary Figure S1. Each cluster’s centroid, which represented the entire cluster, was extracted following clustering. For further molecular dynamics simulation, the compounds of the centroids were utilized.

### 3.5. ADMET Properties

The computational prediction of the ADMET properties (absorption, distribution, metabolism, excretion, and toxicity) of the selected compound was conducted using the SwissADME web tool [43] and ProTox-II server [44].

### 3.6. Molecular Dynamics Simulation

The present investigation employed the Gromacs 2022.4 software [45] to execute an exhaustive 300 ns molecular dynamics (MD) simulation in order to examine the behavior of a protein–ligand complex. We initiated our setup by determining the optimal docking position between the ligand and the protein. In order to define the molecular interactions between the protein and the ligands, the CHARMM36 force field [46] was utilized. The ligands, which comprised our target compound as well as a control binder, were subjected to force-field parameter generation and optimization via the CGneFF server [47]. In order to faithfully simulate electrostatic interactions across a specified distance, the Particle Mesh Ewald (PME) technique [48] was implemented. Subsequently, employing the TIP3P water model, we immersed the system in a receptacle filled with water and introduced Na+ and Cl− ions in an effort to counterbalance its charge [49]. In anticipation of the dynamics simulation, we employed 50,000 iterations of the steepest descent method to alleviate any possible steric collisions and thereby initialize the system. Then, in order to guarantee the stability of our system, we imposed constraints on bond lengths using the LINCS algorithm [50]. Following this, the system was incrementally heated to 310 K during a brief 100 ps simulation using the NVT ensemble (volume, number of particles, and temperature remained constant). The pressure was then adjusted to one atmosphere using the Parrinello–Rahman method during a one-nanometer simulation in the NPT ensemble (temperature, number of particles, and pressure were held constant). During the simulation’s 300 ns production phase, the coordinates of the structure were consistently recorded at 10 ps intervals. In order to establish and maintain a stable simulation environment, we implemented velocity scaling [51] to regulate the temperature and Parrinello–Rahman coupling [52] to regulate the pressure. Ultimately, the system’s conformational stability and fluctuations were evaluated through the utilization of metrics including root mean square deviation (RMSD) and root mean square fluctuation (RMSF). Utilizing GROMACS’ built-in tools, hydrogen bonding patterns were analyzed in order to gain a deeper comprehension of the dynamic interactions occurring within the protein–ligand complex.

MD simulations were conducted for up to 300 ns to ensure sufficient sampling of the ligand–protein interactions and to observe any potential long-term conformational changes or binding site transitions. Extending the simulation to 300 ns provides a more comprehensive understanding of the stability and behavior of the ligand within the protein’s dynamic environment, which is crucial for accurately assessing its potential efficacy as an inhibitor.

### 3.7. PCA and FEL

The trajectory was prepared prior to principal component analysis by removing the periodic boundary requirement. Utilizing the Gmx_covar component of GROMACS, the covariance matrix was computed. To characterize the correlation between the atomic fluctuations of the protein–ligand combination, the covariance matrix is utilized. The gmx analysis function was employed to compute the covariance matrix’s eigenvalues and eigenvectors. The GROMACS utility ‘gmx anaproj’ was utilized in the end to determine the principal component (PC) coordinates for each individual frame. This action was taken in order to display the trajectory on the personal computers. Further, the stepwise command used for the PCA has been mentioned in details in the Supplementary Section S1.

An analysis of the equilibrium state, which is denoted by the lowest points on the Free Energy Landscape (FEL), and the transitional state, which is illustrated by the obstacles on the FEL, can provide significant insights into biological system phenomena including biomolecule recognition, aggregation, and folding [53]. Equation (3) was employed to estimate the energy distribution in order to compute the FEL.

(3) $$∆G\left(X\right)=-kBTlnP(X)$$

The Gibbs free energy is denoted by the symbol G, the Boltzmann constant is represented by kB, T signifies the absolute temperature, X represents the reaction coordinate, and P(X) represents the probability distribution of the system along the reaction coordinate.

### 3.8. MM/GBSA

The MM/GBSA method combines Molecular Mechanics and Generalised Born Surface Area techniques. The method employed to determine the bond strength between a ligand and a receptor, often a protein, is referred to as a strategy for predicting the free energy of binding. The binding free energy of the complex was calculated in the final 20 nanoseconds of the simulation using the MM/GBSA technique and the GROMACS plugin gmx_MMGBSA [54,55]. The analysis of binding free energy was conducted using the MM/GBSA approach. The MM/GBSA calculation was performed using the following equation:

(4) $$∆G=G_{complex}-[G_{receptor}+G_{ligand}]$$

(5) $${\Delta G}_{binding}=\Delta H-T\Delta S$$

(6) $$\Delta H=\Delta G_{GAS}+\Delta G_{SOLV}$$

(7) $$\Delta G_{GAS}=\Delta E_{EL}+\Delta E_{VDWAALS}$$

(8) $$\Delta G_{SOLV}=\Delta E_{GB}+\Delta E_{SURF}$$

(9) $$\Delta E_{SURF}=\Delta .SASA$$

Equation (1) defines ΔG*_complex_* as the total free energy of the protein–ligand complex, ΔG*_receptor_* as the total free energy of the unbound protein, and ΔG*_ligand_* as the total free energy of the ligand in the solvent. The equations within the range of (4)–(9) illustrate the correlation between different components, including solvation free energy change (ΔG_SOLV_), conformational entropy change (−TΔS), enthalpy change (ΔH), and gas-phase energy differential (ΔG_GAS_). The solvent-accessible surface area (SASA) was defined as the surface area accessible to the solvent. Conversely, the letter γ represented the surface tension of the solvent. The ΔE_VDWAALS_ represents alterations in van der Waals energy, while ΔE_EL_ signifies variations in electrostatic energy. ΔE_GB_ signifies changes in solvation energies for polar molecules, while ΔE_SURF_ indicates changes in solvation energies for non-polar substances.

## 4. Conclusions

This study underscores the critical need for new treatments against antibiotic-resistant bacteria, particularly targeting the enzyme NDM-1. The current study employed utilized a combination of pharmacophore-based virtual screening, molecular docking, and molecular dynamics simulations to identify potential inhibitors such as ZINC29142850 (Z1), ZINC78607001 (Z2), and ZINC94303138 (Z3). These compounds demonstrated promising binding affinities and stability, surpassing a control with their effective interactions at key catalytic sites. Notably, Z2 stood out with the highest binding affinity and stability, suggesting its potential as a robust inhibitor. These computational findings highlight the feasibility of using advanced simulation techniques to accelerate drug design, paving the way for experimental validation and further optimization of these candidates. By contributing to the development of effective treatments against multidrug-resistant infections, this study marks a significant step forward in the fight against global antibiotic resistance.

## Figures and Tables

**Table 1:** Screened compounds (66) with their top binding score (BS) and the normalized binding score (BS). Highlighted compounds showed better normalized BS than the control marked in bold.

S.no	Top BS (kcal/mol)	Compound ID	Normalized BS	S.no	Top BS (kcal/mol)	Compound ID	Normalized BS
**1**	−8.884	ZINC14802618	−7.86967	35	−7.131	ZINC29142850	−6.72855
**2**	−8.104	ZINC94303138	−7.79811	36	−7.305	ZINC14265783	−6.72711
**3**	−8.696	ZINC71983666	−7.71111	37	−6.965	ZINC72008621	−6.70222
**4**	−8.296	ZINC02927577	−7.53545	38	−7.233	ZINC11748858	−6.69533
**5**	−8.45	ZINC71986759	−7.50078	39	−7.442	ZINC04851607	−6.69456
**6**	−8.168	ZINC71924775	−7.43711	40	−6.921	ZINC40034395	−6.63233
**7**	−7.715	ZINC71924780	−7.40022	41	−6.824	ZINC78498764	−6.62466
**8**	−8.327	ZINC02927521	−7.39134	**42**	**−7.307**	**control_hydrolyzed-oxacillin**	**−6.59467**
**9**	−7.827	ZINC35548858	−7.29667	43	−6.939	ZINC12384097	−6.56033
**10**	−7.927	ZINC78607001	−7.23967	44	−7.142	ZINC04851208	−6.52722
**11**	−7.496	ZINC32932272	−7.21522	45	−6.751	ZINC39357686	−6.52045
**12**	−8.214	ZINC32932744	−7.19656	46	−7.175	ZINC40868984	−6.51555
**13**	−7.915	ZINC40133417	−7.18156	47	−6.987	ZINC12741918	−6.50422
**14**	−7.529	ZINC11418769	−7.15845	48	−6.894	ZINC58344565	−6.45766
**15**	−7.749	ZINC38697967	−7.12411	49	−7.243	ZINC04851257	−6.45056
**16**	−7.591	ZINC05441336	−7.11533	50	−6.844	ZINC39357685	−6.43023
**17**	−7.578	ZINC05006123	−7.03278	51	−6.813	ZINC32764688	−6.42144
**18**	−7.66	ZINC65610187	−7.03189	52	−7.085	ZINC78444367	−6.41122
**19**	−7.414	ZINC57076878	−7.03111	53	−6.945	ZINC13012826	−6.40767
**20**	−7.499	ZINC24929111	−7.016	54	−6.649	ZINC14481796	−6.32767
**21**	−7.591	ZINC78899383	−7.00111	55	−7.135	ZINC78556987	−6.32434
**22**	−7.381	ZINC12753875	−6.95856	56	−6.918	ZINC05393487	−6.29611
**23**	−7.495	ZINC12360899	−6.95567	57	−7.049	ZINC04851606	−6.29289
**24**	−7.378	ZINC11486594	−6.93356	58	−6.935	ZINC09743679	−6.28045
**25**	−7.708	ZINC78717525	−6.91089	59	−6.642	ZINC38698169	−6.25534
**26**	−7.488	ZINC65610185	−6.90978	60	−6.914	ZINC09743666	−6.24689
**27**	−7.4	ZINC14519389	−6.88178	61	−6.907	ZINC16818978	−6.23034
**28**	−7.156	ZINC11486626	−6.84944	62	−7.147	ZINC02333181	−6.17489
**29**	−7.123	ZINC71881695	−6.77878	63	−6.804	ZINC19526877	−6.11789
**30**	−7.115	ZINC48302108	−6.77722	64	−6.42	ZINC14500916	−6.03978
**31**	−7.605	ZINC06606192	−6.765	65	−6.601	ZINC15419422	−5.98367
**32**	−7.146	ZINC31954796	−6.75566	66	−6.813	ZINC06606196	−5.88189
**33**	−7.155	ZINC04851213	−6.74545	67	−6.292	ZINC05253733	−5.68456
**34**	−7.097	ZINC01028321	−6.736				

**Table 2:** Molecular interactions of compounds with key binding site residues.

Compounds	Conventional Hydrogen Bonds	Carbon-Hydrogen Bonds	Pi-Cation	Pi-Alkyl	Pi-Pi T-Shaped Interaction	Pi-Sigma Bonds
Control	Gln123, His250, Lys211, Asn220, Asp124, His189	His122, Gly219,	Trp93			
Z1	His 189	His122, Gly219, Asp124	Met67, Met154	Ile35, His250	Trp96	
Z2		Asn220	Met67	His122, Val73	Phe70	Ile35
Z3	Gly219, Ser217, His250, Lys211, Lys216, Asp212, Ala74	His189, Val73	Met67, Phe70			

**Table 3:** The ADME-toxicity study of the top three hit compounds.

Molecule	ZINC29142850 (ZN1)	ZINC78607001 (ZN2)	ZINC94303138 (ZN3)
MW	458.53	423.94	839.94
Rotatable bonds	10	7	28
H-bond acceptors	5	2	14
H-bond donors	2	1	6
MR	125.96	125.94	203.12
TPSA	128.45	54.34	300.4
iLOGP	1.49	3.69	0
ESOL class	Soluble	Moderately soluble	Moderately soluble
Lipinski violations	0	0	3
PAINS alerts	0	0	1
Predicted toxicity class	4	4	4

## Data Availability

Data are contained within this article.

## Supplementary Materials

The following supporting information can be downloaded at: https://www.mdpi.com/article/10.3390/ph17091183/s1, Table S1. Binding energies of the L-captopril (PDB ID: 4EXS) and bisthiazolidine (PDB ID: 4U4L) docked with NDM-1 binding pocket; Figure S1. Stepwise screenshot of the steps used for Tanimoto Similarity and Clustering; Figure S2. 3D representation of the interaction between protein and compounds (a, b) Control, (c, d) Z1, (e, f) Z3 and (g, h) Z2; Table S2. Standard deviation (SD) and standard error of the mean (SEM) values for the MMGBSA energy components (EEL, EGB, ESURF, GGAS, GSOLV, and TOTAL) for the control, Z1, Z2 and Z3 compounds; Section S1. Method for PCA.
