# Supplementary material for: Pharmacophore-Based Study: An In Silico Perspective for the Identification of Potential New Delhi Metallo-β-lactamase-1 (NDM-1) Inhibitors
Source: Pharmaceuticals (Basel). 2024 Sep 9;17(9):1183. doi: 10.3390/ph17091183 (PMC11435111; doi:10.3390/ph17091183)
Supplement: Supplementary file 1 [file pharmaceuticals-17-01183-s001.zip › Supplementary.pdf]

Supplementary

Table S1. Binding energies of the L-captopril (PDB ID: 4EXS) and bisthiazolidine (PDB ID: 4U4L) docked with NDM-1 binding pocket

Modes	Binding energy (Kcal/mol) L-captopril	Binding energy (Kcal/mol) Bisthiazolidine
**1**	-4.8	-4.5
**2**	-4.6	-4.4
**3**	-4.4	-4.2
**4**	-4.4	-4.2
**5**	-4.3	-4.2
**6**	-4.3	-4.2
**7**	-4.2	-4
**8**	-4.2	-3.9
**9**	-4.2	-3.9
**10**	-4.2	-3.9
**11**	-4.2	-3.9
**12**	-4.1	-3.8
**13**	-4.1	-3.7
**14**	-4	-3.7
**15**	-4	-3.6
**16**	-4	-3.6
**17**	-4	-3.5
**18**	-3.9	-3.5
**19**	-3.9	-3.4
**20**	-3.9	-3.4

```python
[10]: import numpy as np
import pandas as pd
import matplotlib.pyplot as plt
from sklearn.decomposition import PCA
from sklearn.cluster import KMeans
from scipy.spatial.distance import cdist

pca = PCA(n_components=2)
reduced_data = pca.fit_transform(similarity_matrix)

k = 3
kmeans = KMeans(n_clusters=k, random_state=42)
clusters = kmeans.fit_predict(reduced_data)

centroids = kmeans.cluster_centers_

similarity_matrix['cluster'] = clusters

plt.figure(figsize=(10, 6))
plt.scatter(reduced_data[:, 0], reduced_data[:, 1], c=clusters, cmap='viridis', marker='o', s=50)
plt.scatter(centroids[:, 0], centroids[:, 1], c='red', marker='x', s=100, label='Centroids')
plt.title('K-means Clustering with Centroids')
plt.xlabel('Principal Component 1')
plt.ylabel('Principal Component 2')
plt.legend()
plt.savefig('clustered_image.png')

distances = cdist(reduced_data, centroids, 'euclidean')
closest_indices = np.argmin(distances, axis=0)

closest_molecule_indices = all_indices[closest_indices]

closest_molecule_data = []
for idx in closest_molecule_indices:
    molecule_data = first_molecule_df.loc[first_molecule_df.index == idx].copy()
    closest_molecule_data.append(molecule_data)

closest_molecules_df = pd.concat(closest_molecule_data)

print("Closest molecule data to each centroid:")
print(closest_molecules_df)
```

```python
[6]: import numpy as np
import matplotlib.pyplot as plt
import seaborn as sns
all_indices = np.arange(42)
similarity_matrix = first_molecule_df.pivot_table(index="Molecule_Index_1", columns="Molecule_Index_2", values="Tanimoto_Similarity", aggfunc='mean').
plt.figure(figsize=(12, 10))
sns.heatmap(similarity_matrix, cmap="viridis", annot=False, square=True, linewidths=.5, cbar_kws={"shrink": .5})
plt.title('Tanimoto Similarity Heatmap')
plt.savefig('tanimoto.png')
```

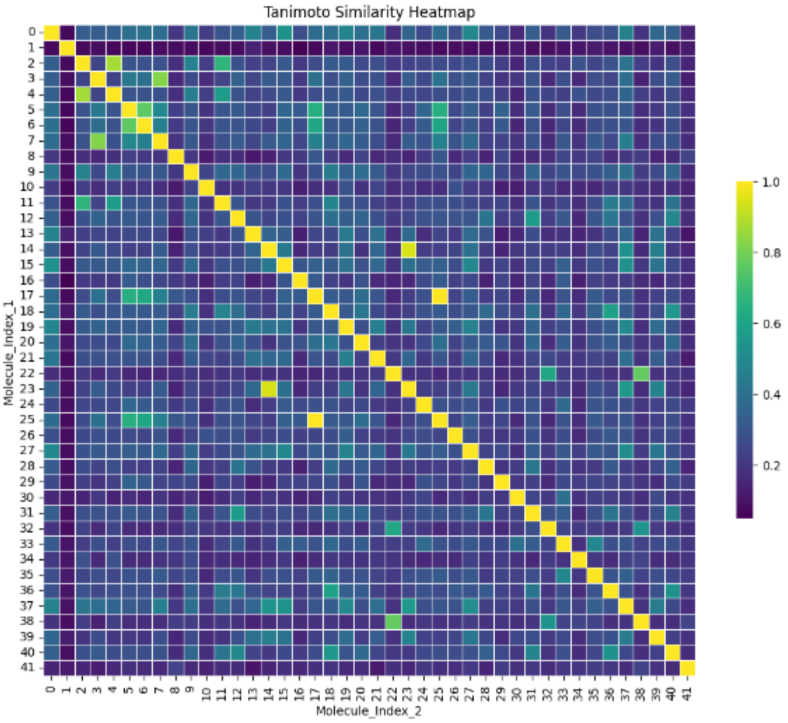

Figure S1. Stepwise screenshot of the steps used for Tanimoto Similarity and Clustering

S1. Method for PCA

Principal Component Analysis (PCA) in GROMACS involves a few steps. Below is a stepwise guideline along with explanations for each command used.

Step 1: Generate the Covariance Matrix

The first step in PCA is to compute the covariance matrix, which captures the variations in atomic positions during the simulation.

Command:

Perform covariance analysis

echo "1\n3" | gmx covar -s md.tpr -f md_NJ.xtc -o eigenvalues.xvg -n index.ndx -v eigenvectors.trr -xpma covapic.xpm

Step 2: Perform PCA

After calculating the covariance matrix, the next step is to analyze the eigenvectors to determine the principal components.

Command:

Perform PCA (Principal Component Analysis)

echo "1\n3" | gmx anaeig -s md.tpr -f md_NJ.xtc -v eigenvectors.trr -n index.ndx -first 1 -last 2 -2d pca.xvg

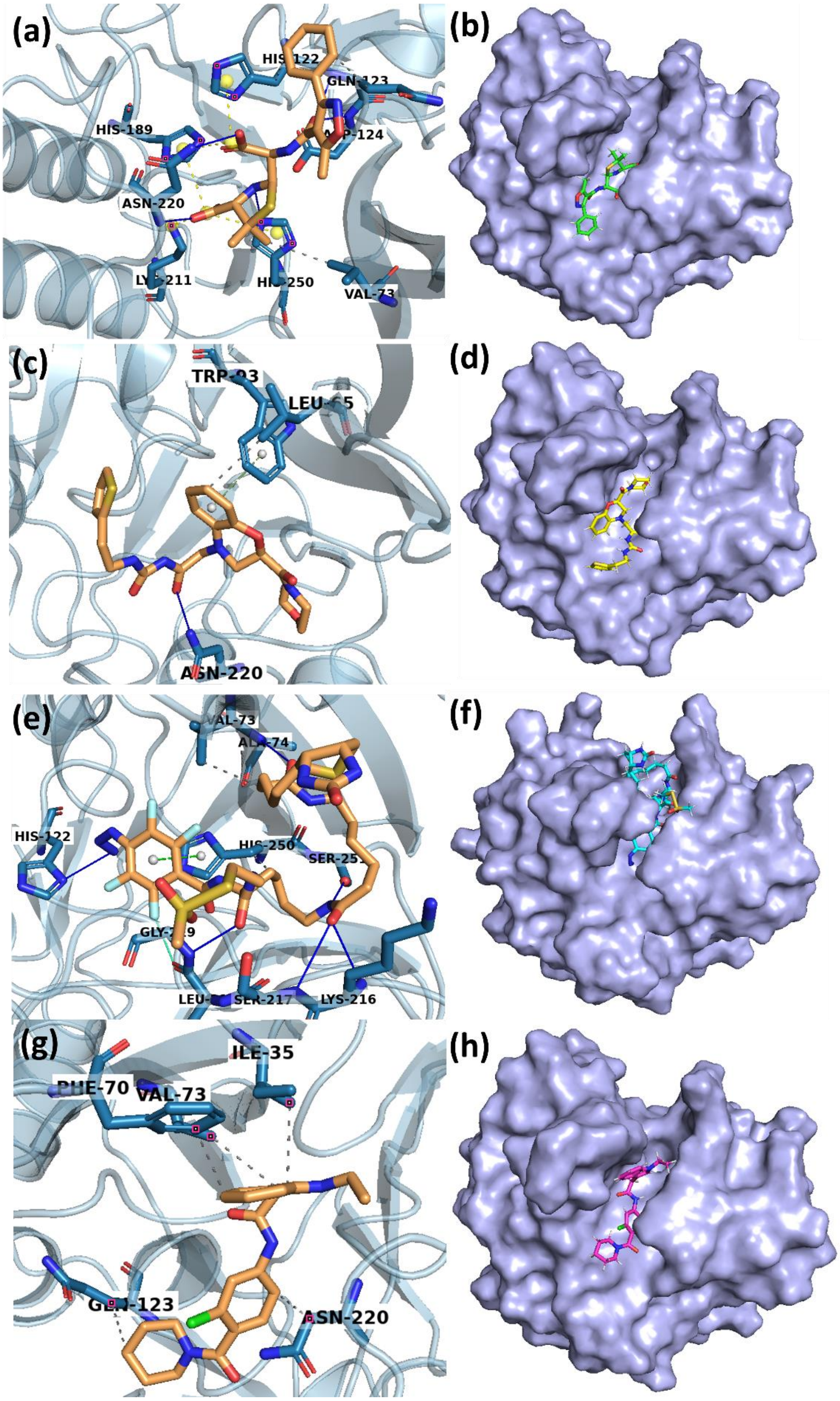

Figure S2. 3D representation of the interaction between protein and compounds (a, b) Control, (c, d) Z1, (e, f) Z3 and (g, h) Z2.

Table S2. Standard deviation (SD) and standard error of the mean (SEM) values for the MMGBSA energy components (EEL, EGB, ESURF, GGAS, GSOLV, and TOTAL) for the control, Z1, Z2 and Z3 compounds.

	**Control**		**Z1**		**Z2**		**Z3**	
	SD	SEM	SD	SEM	SD	SEM	SD	SEM
**EEL**	4.94	0.69	7.45	1.33	11.48	1.61	9.73	1.36
**EGB**	5.08	0.71	7.08	1.14	8.85	1.24	9.83	1.38
**ESURF**	0.43	0.06	0.64	0.07	0.34	0.05	1.04	0.15
**GGAS**	6.18	0.87	10.20	1.77	11.40	1.60	13.50	1.89
**GSOLV**	4.94	0.69	7.49	1.29	8.85	1.24	9.13	1.28
**TOTAL**	3.25	0.45	3.50	0.49	3.42	0.48	5.95	0.83
